# Krill Oil Supplementation and Muscle Health in Older Age: Broad Benefits Without Boundaries?

**DOI:** 10.1016/j.jnha.2025.100767

**Published:** 2025-12-26

**Authors:** Riccardo Calvani, Emanuele Marzetti, Anna Picca

**Affiliations:** a Department of Geriatrics, Orthopedics and Rheumatology, Università Cattolica del Sacro Cuore, Rome, Italy; b Fondazione Policlinico Universitario “Agostino Gemelli” IRCSS, Rome, Italy; c Department of Medicine and Surgery, LUM University, Casamassima, Italy

The progressive decline of muscle mass, strength, and power observed with advancing age, is a major public health concern [1]. Identifying accessible interventions, including nutrition, to support muscle health in older adults has therefore become a scientific and clinical priority [2].

Through a secondary analysis of a randomized controlled trial [3], Hayman et al. [4] explored whether the effects of krill oil supplementation on muscle function and size differed according to sex, age, or body mass index (BMI) in adults aged ≥65 years. Participants were randomized to receive either 4 g/day of krill oil or an isocaloric control oil for six months. Outcome measures included knee extensor strength, grip strength, vastus lateralis muscle thickness assessed by ultrasonography, and surface electromyography parameters. The authors report that improvements in muscle strength and muscle size following krill oil supplementation were comparable across sexes, age groups, and BMI categories. These findings expand on the original trial indicating that long-chain omega-3 polyunsaturated fatty acids (LCn-3 PUFA) from krill oil can increase muscle strength and thickness in older adults [3].

These results contribute interesting insights to a rapidly evolving field. Nutritional interventions are often planned concomitant to exercise to produce measurable muscle benefits. Early mechanistic studies showed that LCn-3 PUFA increases muscle protein synthesis rates in older adults [5], and longer-term fish-oil supplementation increases muscle mass and grip strength without exercise training [6]. However, several trials combining LCn-3 PUFA with resistance training reported potential sex-specific variations, where women appear to derive larger functional benefits from supplementation than men [7]. Against this drawback, the current analysis provides evidence that krill oil supports improvements in muscle size and strength in both sexes in the absence of structured exercise.

Although men and women experienced similar gains in strength and muscle thickness, changes in neuromuscular membrane excitability (M-wave amplitude) differed by sex. Men receiving krill oil showed increases in M-wave amplitude whereas no change was observed in women. While similar bioavailability was evident through comparable changes in omega-3 index between sexes in the intervention group [3], sex-specific neuromuscular effects suggest potential mechanistic differences in how LCn-3 PUFA influence the neuromuscular system. Whether such differences are mediated by hormonal factors, lipid membrane characteristics, or neural recruitment patterns remains unknown and warrants further mechanistic investigation.

Equally relevant is the finding that muscle strength and thickness gains did not translate into improvements in performance-based function (chair-rise time or 4-m walk test). However, this should not overshadow the biological relevance of the intervention. Ceiling effects are commonly reported in community-dwelling adults with high baseline function, limiting the sensitivity of short-timed tests to detect changes in the absence of task-specific exercise [8]. Indeed, functional gains often require both physiological improvements and behavioral training to be transferred into daily mobility.

Another relevant aspect of the study by Hayman et al. [4] is the comparison across BMI categories (i.e., <25 kg/m^2^ vs. ≥ 25 kg/m^2^). Overweight and obesity are associated with impaired muscle metabolism and lipid-driven anabolic resistance, which might theoretically reduce responsiveness to omega-3 supplementation. Yet, krill oil improved muscle outcomes regardless of BMI classification, suggesting its effects are robust to metabolic variability. This broad applicability warrants further investigation as it may underscore krill oil as a potentially scalable intervention for muscle health in diverse older adult populations.

While this secondary analysis was not powered for subgroup interactions, an important limitation acknowledged by the authors, the absence of differential effects across age and sex groups should be viewed as a promising basis for generalizable intervention strategies rather than a null finding. Future trials specifically powered to explore subgroup effects will help strengthen inference and investigate whether the observed sex-specific neuromuscular adaptations hold under varied training conditions or over longer supplement durations.

In conclusion, krill oil supplementation may represent a practical, biologically meaningful strategy to support muscle strength and size in older adults with benefits arising irrespective of sex, age, or BMI. As healthcare systems confront growing demands related to mobility impairment in aging populations, accessible interventions such as LCn-3 PUFA supplementation deserve attention, particularly in combination with exercise strategies that may amplify and translate physiological gains into functional outcomes. Even a handful of benefit deserves serious inquiry when it comes to preserving autonomy in later life.

## Declaration of Generative AI and AI-assisted technologies in the writing process

No Generative AI or AI-assisted technologies were used in the writing process.

## Funding

This work was partly supported by intramural research grant from the Università Cattolica del Sacro Cuoreto E.M. (D1.2024 and D1.2025), the Italian Ministry of Health(Ricerca Corrente 2025), and the nonprofit research foundation “Centro Studi Achille e Linda Lorenzon”(N/A). This work was also partly supported by Next Generation EU PRIN 2022YNENP3 to E.M. E.M. and R.C. acknowledge co-funding from Next Generation EU, in the context of the National Recovery and Resilience Plan, Investment PE8—Project Age-It: “Ageing Well in an Ageing Society”. This resource was co-financed by the Next Generation EU (DM 1557 11.10.2022). The views and opinions expressed are only those of the authors and do not necessarily reflect those of the European Union or the European Commission. Neither the European Union nor the European Commission can be held responsible for them.

## Declaration of competing interest

EM and RC report financial support was provided by the Italian Ministry of Health and the European Commission. AP has no known competing financial interests or personal relationships that could have appeared to influence the work reported in this paper.
